# Physical activity and Mediterranean diet as potential modulators of osteoprotegerin and soluble RANKL in g*BRCA1/2* mutation carriers: results of the lifestyle intervention pilot study LIBRE-1

**DOI:** 10.1007/s10549-021-06400-7

**Published:** 2021-09-27

**Authors:** Leonie Neirich, Maryam Yahiaoui-Doktor, Jacqueline Lammert, Maryam Basrai, Benjamin Seethaler, Anika Berling-Ernst, Juliane Ramser, Anne S. Quante, Thorsten Schmidt, Uwe Niederberger, Kerstin Rhiem, Rita Schmutzler, Christoph Engel, Stephan C. Bischoff, Martin Halle, Marion Kiechle, Sabine Grill

**Affiliations:** 1 Department of Gynecology and Center for Hereditary Breast and Ovarian Cancer, University Hospital Rechts der Isar, Technical University of Munich (TUM), Munich, Germany; 2 Institute for Medical Informatics, Statistics and Epidemiology (IMISE), University of Leipzig, Leipzig, Germany; 3 Institute of Nutritional Medicine, University of Hohenheim, Stuttgart, Germany; 4 Department of Prevention, Rehabilitation and Sports Medicine, Faculty of Medicine, University Hospital Rechts der Isar, Technical University of Munich (TUM), Munich, Germany; 5 Comprehensive Cancer Center, University Hospital Schleswig-Holstein, Kiel, Germany; 6 Institute for Medical Psychology and Medical Sociology, University Hospital Schleswig-Holstein, Kiel, Germany; 7 Center for Hereditary Breast and Ovarian Cancer, University Hospital Cologne, Cologne, Germany; 8 DZHK (German Centre for Cardiovascular Research), Partner Site Munich Heart Alliance, Munich, Germany; 9 Institute of Human Genetics, Medical Center - University of Freiburg, Faculty of Medicine, University of Freiburg, Freiburg, Germany

**Keywords:** *BRCA1/2* mutation carriers, Breast cancer, OPG, RANKL, Lifestyle intervention, Physical activity, Mediterranean diet, Fatty acids

## Abstract

**Purpose:**

Emerging evidence suggests that the progesterone-mediated receptor activator of nuclear factor κB (RANK)/soluble RANK ligand (sRANKL)/osteoprotegerin (OPG) pathway plays an important role in mammary carcinogenesis and is hyperactivated in germline (g)*BRCA1/2* mutation carriers. We analyzed the effects of a 3-month intensive lifestyle intervention within the LIBRE-1 study on the serum levels of OPG and sRANKL and hypothesized that the intervention program provides a beneficial impact on the biomarkers by increasing OPG and reducing sRANKL serum concentrations.

**Methods:**

Serum levels of OPG and sRANKL of 49 g*BRCA1/2* mutation carriers were quantified using enzyme-linked immunosorbent assays. We used previously collected blood samples from participants of the prospective LIBRE-1 study, who were randomized into an intervention group (IG), increasing physical activity and adherence to the Mediterranean diet (MedD) through supervised sessions from study entry to the first study visit after 3 months and a usual-care control group (CG). Differences in biomarker levels before and after the 3-month intervention were tested within and between study groups.

**Results:**

The lifestyle intervention resulted in a significant increase in OPG for participants in both the IG (*q* = 0.022) and CG (*q* = 0.002). sRANKL decreased significantly in the IG (*q* = 0.0464) and seemed to decrease in the CG (*q* = 0.5584). An increase in the intake of Omega-3 polyunsaturated fatty acids was significantly associated with an increase in OPG (*r* = 0.579, *q* = 0.045). Baseline serum levels of sRANKL were a strong predictor for the change of sRANKL in the course of the intervention (ß-estimate = − 0.70; *q* = 0.0018). Baseline physical fitness (assessed as VO_2_peak) might predict the change of OPG in the course of the intervention program (ß-estimate = 0.133 pg/ml/ml/min/kg; *p* = 0.0319; *q* = 0.2871).

**Conclusion:**

Findings from this pilot study seem to confirm our hypothesis by showing an increase in OPG and decrease in sRANKL over a 3-month lifestyle intervention and suggest that increased physical activity and adherence to the MedD are potent modulators of the biomarkers OPG and potentially sRANKL.

**Supplementary Information:**

The online version contains supplementary material available at 10.1007/s10549-021-06400-7.

## Background

Women with a germline mutation in the tumor suppressor genes *BRCA1/2* have a high lifetime risk of developing breast cancer (BC) or ovarian cancer (OC) (69–72% and 16–59%, respectively) [1]. Since the penetrance of cancer disease is high but incomplete, the existence of risk-modulating factors has been postulated, some of which could be lifestyle-associated [2, 3]. While scientific research has demonstrated that lifestyle factors, such as physical activity and nutrition, are modifiers of risk for sporadic BC [4–6], the impact on genetically predisposed women, such as g*BRCA1/2* mutation carriers, is still unclear. At present, prophylactic mastectomy and/or salpingoovarectomy are the only available, but rather drastic primary prevention options for these women, presenting an urgent need for developing less invasive strategies [7].

With regards to the potential of chemoprevention, current findings suggest that the osteoprotegerin (OPG)/receptor activator of nuclear factor (NF)-κB (RANK)/ RANK ligand (RANKL) pathway, a key regulator in bone metabolism, plays a crucial role in the tumorigenesis of g*BRCA1/2*-associated BC [8–13]. The activation of the NF-κB pathway through progesterone-dependent RANK/RANKL signaling leads to breast tissue proliferation [14–16], as well as the induction of BC [17, 18], while genetic and pharmacological RANK inhibition evidently reduce the likelihood of tumor formation [13]. The RANK/RANKL cascade might provide potential targets for chemoprevention for g*BRCA1/2*-associated BC [19], especially since the pathway seems hyperactivated in g*BRCA1/2* mutation carriers leading to massively elevated RANKL expression [20–22] as well as notably lower levels of serum OPG [23–26]. Functioning as a decoy receptor for RANKL, lower OPG levels lead to a weaker RANKL inhibition, resulting in up to 75% higher BC risk for g*BRCA1* mutation carriers with below-average OPG levels compared to high-risk patients with above-average serum levels [26].

These abnormal molecular patterns in g*BRCA1/2* mutation carriers seem highly relevant in developing disease prevention strategies, as physical activity has been found to affect serum levels of OPG and possibly sRANKL. Studies in the general population suggest that endurance training increases circulating levels of OPG [27, 28] and might diminish serum levels of progesterone [29, 30] and RANKL [27].

Additionally, there is a growing body of evidence that the Mediterranean diet (MedD) might modulate these biomarkers [31, 32]. The MedD describes a nutritional concept that is based on vegetables, grains, and nuts and is characterized by a proportionally high intake of olive oil and fish, which are rich in the n-3 and n-9 polyunsaturated fatty acids (PUFA), as well as a moderate intake of dairy products and low consumption of red meat, which contain primarily n-6 PUFA [33]. Several studies have shown that adherence to the MedD can reduce the risk of cardiovascular disease [34], inflammation [35], as well as mortality due to BC [36].

Using data from the LIBRE-1 study cohort, we aimed to explore the effects of a structured lifestyle intervention program on biomarkers that seem to be dysregulated in g*BRCA1/2* mutation carriers [37, 38].

## Subjects and methods

### Study cohort and participant recruitment

**Fig. 1:**
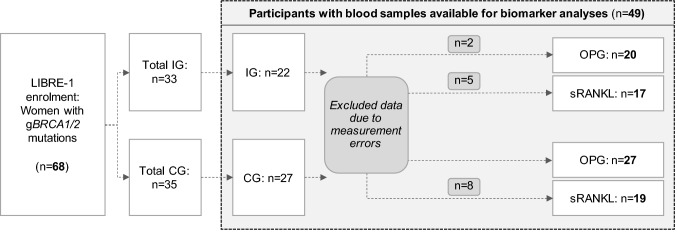
LIBRE-1 study population flow chart showing initial numbers of LIBRE-1 participants and available participant data after data cleansing. *BRCA1/2* breast cancer genes 1 and 2; *CG* control group; *IG* intervention group; *OPG* osteoprotegerin; *sRANKL* soluble receptor of nuclear factor κB ligand

Women aged 18–69 years with a confirmed germline mutation in the *BRCA1* or *BRCA2* genes were eligible to participate in the LIBRE-1 study, a multicenter, prospective, two-armed randomized (1:1) controlled lifestyle intervention study. The LIBRE-1 study started in February 2014 [37] and was planned as the pilot study of the subsequently performed larger LIBRE-2 study, which started in 2015 [38]. The LIBRE-2 study is currently still recruiting (*n* = 484, as of May 2021). The primary endpoint of the LIBRE-1 study was to investigate the feasibility of a structured lifestyle intervention and its acceptance by the participants, which were both confirmed positive. [37, 39]. In the present secondary analysis of the LIBRE-1 cohort, we analyzed OPG and sRANKL serum levels from all participants of the LIBRE-1 study (total *n* = 68) of whom both blood samples and all clinical data, including questionnaires, anthropometry, cardiopulmonary exercise testing (CPET), and measures on fatty acid profiles in red blood cell membranes (RBCM), were available for both time points of study entry (SE) as well as 3 months later (V1), resulting in 49 individuals (see Fig. 1). We focused on the 3-month period of the LIBRE-1 intervention as more pronounced lifestyle-associated effects on the biomarkers were expected during the intensive phase of the program [40]. Also, because of the small sample size and limited detection capacities of our quantitative tests, smaller changes in OPG and sRANKL serum levels might be more difficult to detect. Both g*BRCA1/2* mutation carriers with a history of BC or OC prior to SE (*diseased* participants) and *non-diseased* participants, without previous BC or OC, were included in the LIBRE-1 study. Informed written consent was obtained from all participants. The study was approved by the ethics committees of the three participating centers (Munich REF: 5686/13, Cologne REF: 13-053, Kiel REF: B-235/13). The LIBRE-1 cohort was recruited from three German university hospitals in Munich, Kiel, and Cologne, members of the German Consortium for Hereditary Breast and Ovarian Cancer.

### Intervention program

The intervention included a structured endurance training and nutrition education based on the MedD. Over 3 months, the IG received two supervised and one home-based physical training session per week with the objective of increasing physical activity to at least 18 metabolic equivalents of task/-hour, mainly through endurance-based exercise to improve cardiopulmonary fitness. Nutrition education followed a group-based approach on fortnightly basis. The CG attended only one information session at SE on the benefits of physical activity and nutritional recommendations in accordance with the German Nutrition Society [37].

### Assessment of clinical data and questionnaires

Data were collected by means of clinical interviews and questionnaires, CPET, and blood sampling during the study visits at SE and at 3 months (V1) and secured by each study center in a central database using OpenClinica (Waltham, MA, USA). To gain objective data on the effects of the intervention program on cardiopulmonary fitness, the maximal oxygen uptake (VO2 peak in ml/min/kg) as a validated surrogate parameter for endurance performance [41] was measured using CPET. CPET describes a ramp protocol, which measures respiratory gases under gradually increasing resistance to achieve physical exhaustion [42]. Body Mass Index (BMI, kg/m^2^) was determined during physical examination. The Mediterranean Diet Adherence Screener (MEDAS) [43] was used to score the nutritional adherence to the MedD with 14 items on food consumption and MedD habits. To reduce the bias of unanswered questions, the score was calculated as a percentage of positively answered questions (1 = stronger adherence to MedD, 0 = less adherence to MedD) to all answered questions [44]. Participants fasted a minimum of 12 h prior to each study visit. Blood samples were processed to aliquots and either analyzed within 48 h or stored at − 80 °C until usage. Furthermore, we used data of PUFA in the RBCM as well as in plasma that were conducted on the same LIBRE-1 blood samples, as described elsewhere [44].

### Enzyme-linked immunosorbent assays

A total of 98 blood samples from 49 LIBRE-1 g*BRCA1/2* mutation carriers derived from time points SE and V1 were assayed for serum levels of sRANKL and OPG. For quantitative testing of total sRANKL (REF: K1016) and OPG (REF: K1011), we used enzyme-linked immunosorbent assay (ELISA) kits from Immundiagnostik AG (Bensheim, Germany). All tests were conducted according to the manufacturer’s protocol. All samples were analyzed in double measurement on the same microtiter plate. As provided by the manufacturers, 6 standards and 2 control samples for sRANKL and 6 standards and 1 control sample for OPG were run within each kit. In total we used 6 kits for sRANKL and 4 kits for OPG. For quality control, we assessed the intra- and inter-assay coefficients of variation (CV) for each kit, being between 5.6 and 6.5% (inter-assay CV), as well as between 3.5 and 12.6% (intra-assay CV), which is generally accepted as good [45]. All standards and controls were within the default range, as stated by the manufacturer, with the exception that in one sRANKL ELISA run the low control was 6% above the reference range.

### Statistical analysis

Statistical analysis was conducted using GraphPad Prism version 8.4.3. for MacOS (GraphPad Software, San Diego, California, USA). For sRANKL, we excluded data from 13 participants due to technical measurement errors, as serum levels were below the detection rate of the ELISA test. For OPG, we excluded data from 2 participants due to a technical measurement error (no concentrations were provided by the ELISA kits). We thus had data available from 20 participants in the IG and 27 participants in the CG for OPG as well as from 17 participants in the IG and 19 participants in the CG for sRANKL (Fig. 1).

To test for normal distribution, we used the Shapiro–Wilk test [46] as well as histograms for graphic estimates. sRANKL as one of the key parameters in this analysis did not show normal distribution; therefore, to remain consistent, we used non-parametric tests and presented all metric data as median and IQR. To test whether two independent groups were statistically different, we used the Mann–Whitney *U* test. For categorical variables, we used the Fisher’s exact test. To test for differences within each study group before and after the 3-month intervention phase (ΔV1-SE), we used the Wilcoxon matched-pairs signed-rank test. Associations between two variables were tested using Spearman’s rank correlation coefficient (*r*); associations between more than two variables were tested using multiple linear regressions. Multiple testing was adjusted with a false discovery rate of 5% according to Benjamini and Hochberg [47]. An adjusted *p* value (*q*) < 0.05 was considered as statistically significant.

Some sRANKL concentrations yielded a strong positive deviation from the median, resulting in large variances. We conducted statistical outlier analyses using the Tukey method for Boxplots [48], identifying 8 data sets that could be regarded as outliers. However, we decided not to exclude these data for several reasons: firstly, no clinical reference values are yet available for the biomarkers in the context of hereditary BC, not allowing assumptions with regard to clinical implausibility. Secondly, we did not find an indication for measurement errors; hence, by including all measurable concentrations, we aimed at preventing arbitrariness. Thirdly, the comparison of data sets when excluding outliers achieved similar results.

## Results

### Study population

Regarding all participant characteristics included in the present analysis, the study groups IG and CG did not differ significantly at SE (Table 1). To test whether our subgroup of 49 individuals differed from the total LIBRE-1 cohort (*n* = 68) in any of the characteristics shown in Table 1, we performed Mann–Whitney *U* tests between these cohorts, which showed no statistically significant results (data not shown).

**Table 1:** Participant characteristics at study entry (SE)

Participant characteristics	IG (*n* = 22)	CG (*n* = 27)	Total (*n* = 49)
g*BRCA1* mutation, *n* (%)	15 (68%)	17 (63%)	32 (65%)
g*BRCA2* mutation, *n* (%)	7 (32%)	10 (37%)	17 (35%)
Age, *y*	41 (33–48)	43 (35–50)	41 (34–49)
Prior BC or OC disease, *n* (%)^a^	15 (68%)	17 (63%)	32 (65%)
Postmenopausal, *n* (%)^b^ Ever took HRT, *n* (%)^c^ Ever took contraceptives, *n* (%)^d^	11 (50%) 2 (9%) 19 (86%)	14 (52%) 5 (19%) 27 (100%)	25 (51%) 7 (14%) 46 (94%)
BMI > 24.9, kg/m^2^*, n *(%)	8 (36%)	11 (41%)	19 (39%)
Ever smoked, *n *(%)	14 (64%)	17 (63%)	31 (63%)
Pregnancy, *n *(%)^e^ Breastfeeding, *n* (%)^f^	15 (68%) 12 (55%)	18 (67%) 15 (56%)	33 (68%) 27 (55%)
VO_2_ peak, ml/min/kg^g^	23 (21–28)	28 (22–32)	26 (22–31)
MEDAS score, %^h^	46 (36–64)	36 (29–50)	46 (36–64)
OPG, pg/ml	59 (44–71)^i^	54 (40–70)	57 (43–70)^j^
sRANKL, ng/ml	126.4 (39.7–5673.9)^k^	176.4 (52.2–2341.9)^l^	152.5 (44.1–422.8)^m^

There were no differences between the study arms in any of the characteristics considered

Values are medians and interquartile ranges. Continuous variables were compared using the Mann–Whitney *U* test. Categorical variables were compared using the Fisher's exact test. Multiple testing was adjusted with a false discovery rate (FDR) of 5% based on Table 1 resulting in no significant discoveries

*BMI* body mass index; *BC* breast cancer; *CG* control group; *HRT* hormone replacement therapy; *IG* intervention group; *OPG* osteoprotegerin; *OC* ovarian cancer; *sRANKL* soluble receptor of nuclear factor κB ligand

^a^Disease status (cancer type and date of diagnosis)

^b^Postmenopausal status (date of last menstrual period)

^c^Previous intake of hormone replacement therapy for any period longer than 1 month (yes/no)

^d^Previous intake of hormonal contraceptives for any period longer than 1 month (yes/no)

^e^Full-term pregnancies (excluding abortions) (yes/no)

^f^Breastfeeding periods longer than 1 month prior to SE (yes/no)

^g^VO_2_peak (maximal oxygen uptake) measured through cardiopulmonary exercise testing (CPET)

^h^According to the Mediterranean Diet Adherence Screener (MEDAS)

^i^*n*=20

^j^*n*=47

^k^*n*=17

^l^*n*=19

^m^*n*=36

**Fig. 2:**
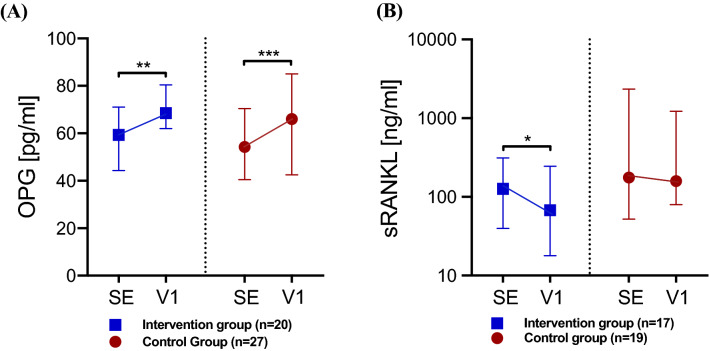
Change in OPG and sRANKL during a 3-month intervention phase for IG and CG. **A** Osteoprotegerin (OPG), **B** soluble receptor of nuclear factor κB ligand (sRANKL). Data are shown as medians with interquartile ranges. Difference to study entry (SE) was tested by the Wilcoxon matched-pairs signed-rank test and is indicated on top of the scatter dot plots by asterisks. Differences in ΔV1-SE between study groups were tested using the Mann–Whitney *U* test. Multiple testing was adjusted with a false discovery rate (FDR) of 5%. Results with an FDR-adjusted *p* value (*q* value) < 0.05 are shown in bold in Table 2. There was no significant difference between IG and CG. *p < 0.0232 (*q* < 0.0464), **p < 0.0073 (*q* < 0.0219), ***p < 0.0004 (*q* < 0.0024). *CG* control group; *IG* intervention group; *V1* 3 months after SE

Individuals from the IG and CG showed a similar significant increase in OPG between SE and V1 (Table 2, Fig. 2A). Furthermore, the IG showed a significant decrease in sRANKL, while the decrease in this biomarker was non-significant for the CG (Table 2, Fig. 2B). The extent to which OPG increased and sRANKL decreased was similar in the IG and CG.

**Table 2:** Within and between group differences in OPG and sRANKL during the course of the 3-month intervention phase (ΔV1-SE)

	IG				CG				
	SE	V1	ΔV1-SE	Within group difference *p* (*q*)	SE	V1	ΔV1-SE	Within group difference *p* (*q*)	Between group difference in Δ *p* (*q*)
OPG (pg/ml) (IG: *n* = 20, CG: *n* = 27)	59.4	68.5	+ 9.1	0.0073 (**0.0219**)	54.2	66.0	+ 11.8	0.0004 (**0.0024**)	0.8230 (0.8230)
sRANKL (ng/ml) (IG: *n* = 17, CG: *n* = 19)	126.4	67.6	− 58.8	0.0232 (**0.0464**)	176.4	159.2	− 17.2	0.4653 (0.5584)	0.3626 (0.5439)

Difference in OPG and sRANKL assessed at study entry (SE) and after the 3-month intervention program was tested using the Wilcoxon matched-pairs signed-rank test. Difference in the change over time (ΔV1-SE) of OPG and sRANKL between study groups was tested using the Mann–Whitney *U* test. Multiple testing was adjusted with a false discovery rate (FDR) of 5% based on Table 2. Results with an FDR-adjusted *p* value (*q* value) < 0.05 are shown in bold

*CG* control group; *IG* intervention group; *OPG* osteoprotegerin; *sRANKL* soluble receptor of nuclear factor κB ligand; *V1* 3 months after SE

**Fig. 3:**
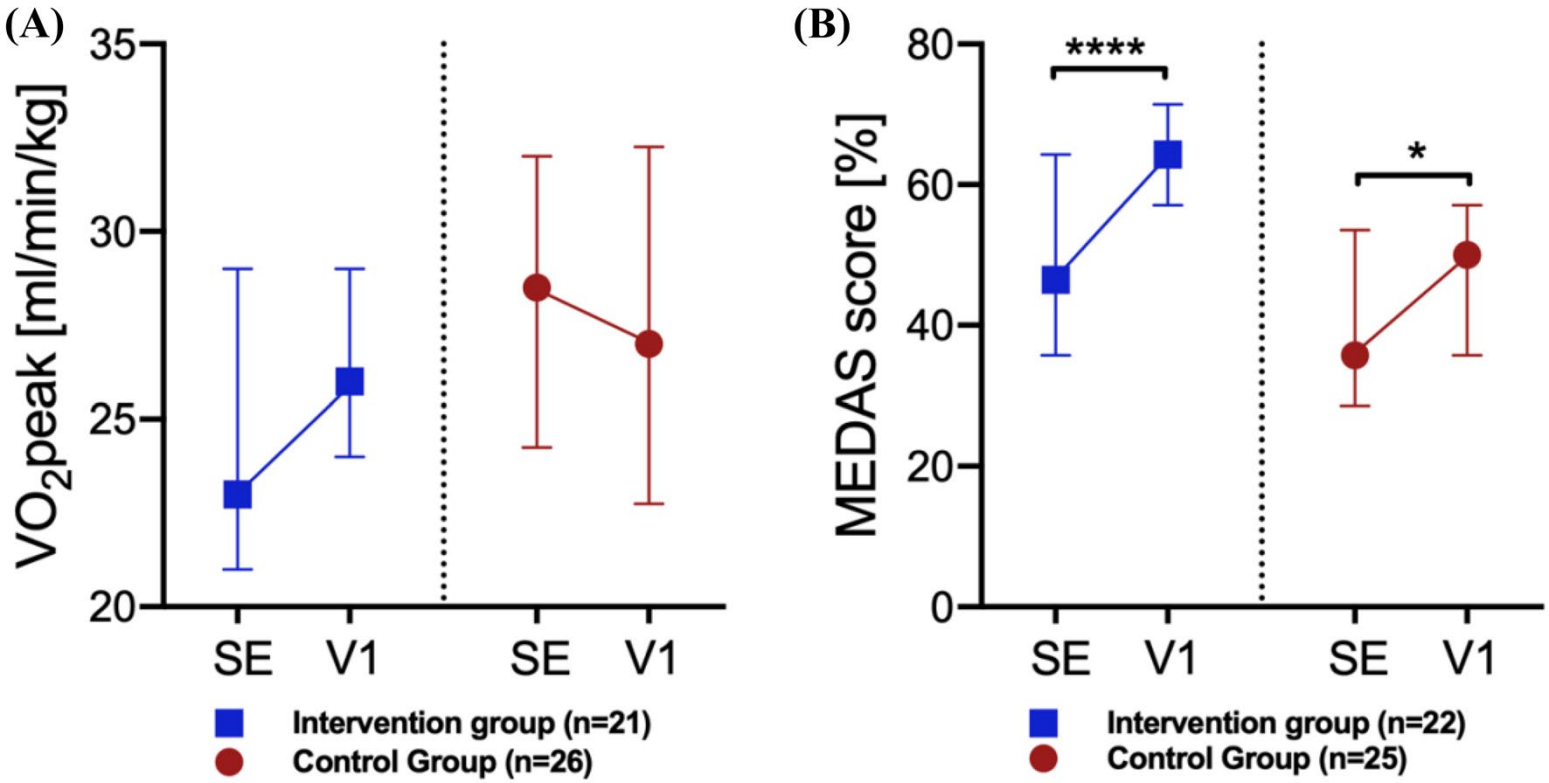
Change in VO_2_peak and MEDAS score during a 3-month intervention phase for IG and CG. **A** Maximal oxygen uptake (VO_2_peak), **B** Mediterranean Diet Adherence Screener (MEDAS) score. Data are shown as medians with interquartile ranges. Difference to study entry (SE) was tested by the Wilcoxon matched-pairs signed-rank test and is indicated on top of the scatter dot plots by asterisks. Differences between study groups were tested using the Mann–Whitney *U* test. Multiple testing was adjusted with a false discovery rate (FDR) of 5% based on Fig. 3. Both IG and CG increased the MEDAS score significantly from SE to V1. *p (*q*) < 0.0165, ****p < 0.0001 (*q* < 0.0002). *CG* control group; *IG* intervention group; *V1* 3 months after SE

For VO_2_peak, while non-significant within and between groups, the IG showed an improvement in VO_2_peak while the parameter in the CG stagnated from SE to V1 (Fig. 3A). Both groups (IG: *p* = 0.0001; *q* = 0.0002), CG: *p* = *q* = 0.0165) similarly increased their MEDAS scores significantly from SE to V1 (Fig. 3B).

**Fig. 4:**
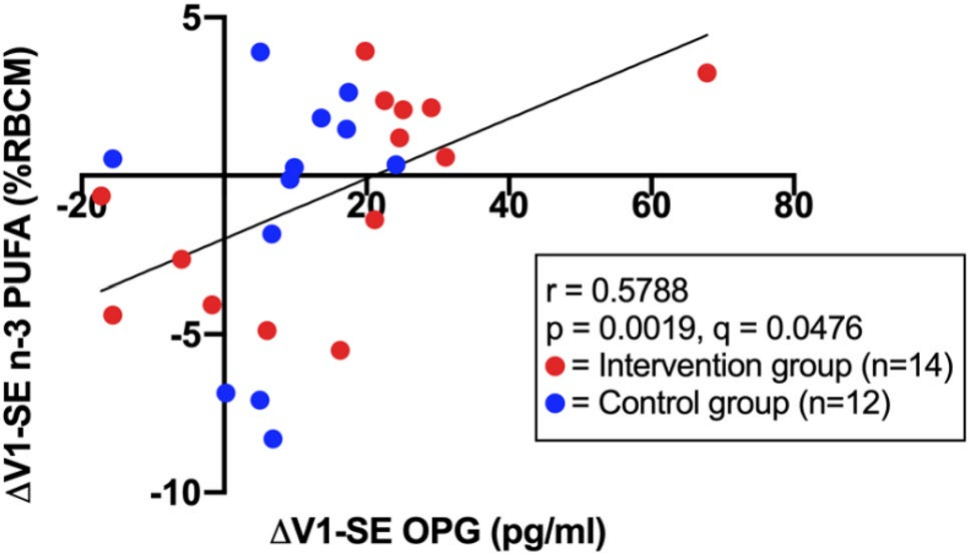
Association between changes in osteoprotegerin (OPG), indicated in the x-axis, and changes in n-3 polyunsaturated fatty acids (PUFA), indicated in the y-axis, over a 3-month intervention phase from SE to V1 (Δ) on a total of *n* = 26 participants of the IG and CG. n-3 PUFA shown as percentage of red blood cell membrane (RBCM) for *n* = 26 participants of the IG and CG. Correlation analyses were conducted using Spearman’s rank correlation coefficient (*r*). Multiple testing was adjusted with a false discovery rate (FDR) of 5% based on *p* values in supplementary table 1, resulting in the *q* values shown. *PUFA* polyunsaturated fatty acid; *n-3* omega-3 fatty acid; *SE* study entry; *V1* 3 months after SE

The correlation analyses of PUFA with the biomarker included *n* = 26 participants from both study groups, as PUFA analyses were only available for these participants. The correlation analyses showed that an increase in total n-3 PUFA in RBCM was significantly correlated with an increase in OPG (Fig. 4), as well as an increase in the n-3 EPA with an increase in OPG (Supplementary Table 1). When looking at the study groups individually, an increase of n-3 PUFA correlated with an increase in OPG in the IG, yet the correlation was not statistically significant after FDR correction (*r* = 0.6176; *p* = 0.0212; *q* = 0.1201). There was no significant association between n-3 PUFA and OPG in the CG (*r* = 0.4545; *p* = 0.1404; *q* = 0.3978).

There was no significant correlation between the changes in n-3, n-6, and n-9 PUFA measured in RBCM or plasma and the changes in sRANKL (Supplementary Table 1). Before FDR correction, both the total n-6 PUFA and oleic acid showed significant associations to sRANKL and OPG when measured in plasma. While an increase in total n-6 PUFA was positively correlated with an increase in sRANKL and negatively correlated with OPG, oleic acid showed the opposite trend (Supplementary Table 1).

For sRANKL, the explorative multivariate regression models showed a significant association between the baseline serum level and the changes in the biomarker from SE to V1 (Table 3). Furthermore, the analysis indicated that past smoking might be related to a stronger increase in sRANKL, yet this association was only significant before the *p* value was adjusted for multiple testing. For OPG, the multivariate analysis showed a significantly positive relationship between baseline VO_2_peak and changes in OPG, yet this association was no longer statistically significant after adjusting for multiple testing.

**Table 3:** Multivariate linear regression analyses for the influence of selected constitutional parameters on changes (ΔV1-SE) in OPG and sRANKL

Independent variables	Dependent variables			
	ΔV1-SE sRANKL		ΔV1-SE OPG	
	Estimate	*p* (*q*)	Estimate	*p* (*q*)
Randomization to IG	382.1	0.6463 (0.8310)	− 4.87	0.4214 (0.7814)
Age	− 6.82	0.8704 (0.9216)	− 0.27	0.4083 (0.7814)
Premenopausal	1190	0.2213 (0.6638)	4.55	0.5036 (0.8241)
Prior BC or OC disease	− 1191	0.1283 (0.4619)	2.71	0.6252 (0.8310)
BMI at SE	− 78.06	0.4049 (0.7814)	0.93	0.1009 (0.4541)
Ever smoked	1642	0.0815 (0.4541)	− 1.37	0.7957 (0.9216)
VO_2_peak, ml/min/kg	− 65.95	0.4341 (0.7814)	1.33	0.0319 (0.2871)
MEDAS score, %	− 4.4	0.8571 (0.9216)	− 0.01	0.5520 (0.8280)
Serum level at SE	− 0.6977	< 0.0001 (**0.0018**)	0.004	0.9646 (0.9646)
*R*^2^ (adj. *R*^2^)	0.6397 (0.5150)		0.2080 (0.01002)	

Multivariate linear regression analyses for the influence of selected constitutional parameters on changes in OPG and sRANKL during a 3-month intervention phase (ΔV1-SE). Multiple testing was adjusted with a false discovery rate (FDR) of 5% based on *p* values in Table 3, resulting in *q* values, as shown in brackets and highlighted in bold if statistically significant

*BC* breast cancer; *BMI* body mass index; *Estimate* has the unit of the dependent variable divided by the unit of the independent variable; *IG* intervention group; *MEDAS* Mediterranean diet adherence screener; *OC* ovarian cancer; *OPG* osteoprotegerin; *SE* study entry; *sRANKL* soluble receptor of nuclear factor κB ligand; *VO*_*2*_*peak* maximal oxygen uptake; *V1* 3 months after SE

## Discussion

Experimental evidence suggests that the hyperactivation of the RANK/RANKL/OPG pathway plays an important role in the differentiation of the mammary gland [9], stem cell expansion [49], and ultimately in mammary carcinogenesis [10] in g*BRCA1/2* mutation carriers. In the present analysis, we explored the potential effects of a structured lifestyle intervention on OPG and sRANKL serum concentrations and demonstrated that the MedD in combination with increased physical activity may be biomarker modulators. To the best of our knowledge, this is the first time that lifestyle intervention-based alterations in OPG and sRANKL serum concentrations were shown in the context of g*BRCA1/2* mutation carriers.

Several studies propose that OPG can be increased directly through physical activity by the mechanism of mechanical bone stress [40, 50, 51]. While Ziegler et al. showed that OPG concentrations increased immediately after an endurance exercise [27], Bergström et al. found significantly elevated OPG levels after a structured 1-year endurance training [28]. Hence, the RANK/RANKL/OPG pathway seems to be responsive to acute as well as chronic exercise [52]. Our data support these findings by showing a significant increase in OPG serum concentrations over a time span of 3 months, indicating a direct responsiveness of the biomarker to changes in health behavior related to physical activity.

In contrast to OPG, the influence of physical activity on sRANKL concentrations is still unclear [28]. Some studies show that exercise can lower sRANKL serum concentrations, but changes are likely to depend on the intensity of exercise [53, 54]. Ziegler et al. demonstrated that sRANKL decreased significantly only when running a marathon, but not a 15 km distance [27]. Scott et al. reported that while endurance training instantly increased OPG, only high-intensity endurance exercise caused an additional decrease in sRANKL [55]. Our results support these findings, where we showed diminishing sRANKL concentrations within both study groups, but only significantly in the IG (Fig. 2B). This might indicate that only a structured and supervised intervention program like in the LIBRE-1 IG provides the intensity needed to effectively change sRANKL concentrations. However, since the intergroup difference in sRANKL changes was not significant, the LIBRE-1 lifestyle intervention might not be sufficient to significantly lower sRANKL levels. While VO_2_peak increased in the IG and decreased in the CG (Fig. 3A), indeed indicating a measurable improvement in cardiopulmonary fitness through the lifestyle intervention, there is a need for further studies with a more intense exercise design with either more frequent training sessions or higher intensities. After all, it remains unclear whether the diminishing effect of exercise on sRANKL is a direct mechanism or whether it is a result of an increase of inhibitory OPG levels [27].

Apart from exercise, OPG and sRANKL seem to be responsive to dietary habits. Various nutritional studies show a modulating impact of PUFA on these biomarkers [56]. In the present analysis we showed a significant association between the intake of n-3 PUFA, specifically EPA, and OPG (Fig. 4). Our results indicate that a structured nutritional intervention might change PUFA intake patterns sufficiently, as only the IG showed a significant association to n-3 PUFA in subgroup comparison to the CG (albeit the non-corrected *p* value), which supports other PUFA analyses on the LIBRE-1 cohort [44]. Our results are supported by several studies, such as Martin-Bautista et al. who reported an increase in OPG by 18% and decrease in sRANKL by 7% after a 1-year consumption of n-3 PUFA [57]. The latter negative correlation between n-3 PUFA and sRANKL was also described by several other studies [58, 59]; however, our data were not able to replicate these results. This might be due to the rather large variances in our sRANKL data and might be aggravated by our small sample size. For n-6 PUFA, which are consumed less when following the MedD, a negative association to OPG as well as a positive association to sRANKL is described [60]. By diminishing the OPG/sRANKL expression, the intake of n-6 PUFA has been associated with an increased inflammatory and osteoclastic activity and hence risk of cardiovascular diseases and osteoporosis [61]. While being non-significant after adjusting for multiple testing, our correlations show congruent results for n-6 PUFA as well as for the n-9 PUFA oleic acid, for which olive oil is a primary dietary source and is known to inhibit osteoclastogenesis. In summary, our correlation analyses seem to confirm the associations between the different PUFA and OPG and sRANKL that are described by various studies using prospective data of g*BRCA1/2* mutation carriers and suggest that the MedD might modulate the biomarker serum levels through modification of PUFA intake.

Furthermore, sRANKL is reported to depend on the metabolic status and to diminish as a response to a reduction of adipose tissue and thus circulating sex hormones, insulin, proinflammatory cytokines, and the adipocytokine leptin [62, 63]. It has been shown that the MedD can improve the metabolic status [64, 65]; hence the nutritional intervention, measured as a significant increase in the MEDAS score (Fig. 3B), might also explain the observed decrease in sRANKL concentrations in the IG. Pasanisi et al. showed in a similar study on g*BRCA1/2* mutation carriers that a dietary MedD intervention can lead to a reduction of sRANKL through reduced levels of proinflammatory IGF-1 [32].

We performed exploratory multiple linear regression models to examine whether participant characteristics at SE might influence biomarker changes during the study. To the best of our knowledge, this is the first time that the dynamics of changes in OPG and sRANKL are assessed with regards to their initial serum levels. For sRANKL, we identified the baseline level as a significant influence on the change of the serum levels, but not for OPG, suggesting baseline sRANKL levels as a potential candidate to predict the outcome of a 3-month lifestyle intervention. For g*BRCA1/2* mutation carriers with initially high sRANKL concentrations, the biomarker seems to decrease stronger over the intervention phase compared to women with lower initial sRANKL serum levels. The fact that sRANKL is associated with a proinflammatory metabolic status [66], indicates that especially participants with a less active lifestyle might benefit from such an intervention program. In turn, there might be a saturation effect for participants with an already healthy lifestyle and initially low sRANKL levels, leading to a smaller decrease in the biomarker through an additional change in health habits. Hence, initial biomarker levels might also affect the modulative potential of lifestyle changes. Multivariate regression models also showed that cigarette smoking might influence the change of sRANKL in the course of the study. It has been shown that smoking can induce the expression of RANKL mRNA [67] and increases the sRANKL/OPG ratio by promoting inflammatory cytokines [68]. In a large case–control study it was demonstrated that cigarette smoking increased the risk for BC in g*BRCA1/2* mutation carriers [69] for which sRANKL/OPG system might present a molecular mechanism of action. According to our data, baseline physical fitness (assessed as VO_2_peak in CPET) was associated with changes in OPG. The higher the participants’ initial VO_2_peak, as a proxy for cardiopulmonary fitness, the more pronounced OPG levels increased during the study. This finding supports the notion that OPG is primarily responsive to physical activity [27] and might indicate a dose–response relationship between cardiopulmonary fitness and OPG serum concentrations. In all, while our results must be regarded as purely explorative, we aim to confirm these findings using prospective data from the larger LIBRE-2 study.

While biomarker changes were more pronounced in the IG, we did not find significant intergroup differences in biomarker changes in any of our analyses. This suggests that all participants, independent of the study group allocation, seem to have made efforts within the study program to adapt to healthier lifestyle choices. We assume that information exchange between participants due to the open design of the study, as well as possible disappointment within the CG with regards to group allocation, which would be consistent with previous findings from the LIBRE-1 study [38], might explain why some effects that were expected only in the IG also occurred in the CG.

Furthermore, our study cohort was likely to inherit a selection bias toward participants prone to above-average health habits, as it has been shown that a prior cancer disease is likely to have confounding effects on subsequent health behavior [70]. Especially the fact that a g*BRCA1/2* mutation or cancer diagnosis often occurs within the familial surrounding, participants—whether themselves diseased or not—are likely to be sensitized for a more proactive engagement toward lifestyle changes [71]. We also did not control for special dietary habits or sport programs of the participants prior to SE, hence we could not account for potentially confounding and/or prolonging effects of individual health behavior on the biomarkers.

The small sample size of this evaluation represents its main limitation. However, this explorative approach aimed at generating novel knowledge regarding a possible association between lifestyle, the biomarkers OPG and sRANKL, and BC in at-risk individuals. The present analysis was conducted as a secondary analysis and was no initial endpoint of the LIBRE-1 study; therefore, study design and sample size were not established based on statistical measures. Hence, we cannot ensure representativeness of our cohort (*n* = 49) compared to the full LIBRE-1 cohort (*n* = 68) or compared to the combined LIBRE-1 and LIBRE-2 cohort after both trials will be finished (planned *n* = 660). These initial findings can be the basis for future studies comprising larger sample sizes. Specifically, by showing that short-term lifestyle modifications can alter serum levels of the observed biomarkers, the present findings will be the foundation for a subsequent analysis of OPG and sRANKL in the larger LIBRE-2 main study.

In the present analysis we only explored the dynamics of OPG and sRANKL in individuals with a g*BRCA1/2* mutation. While serum levels of OPG and sRANKL in healthy individuals have been evaluated in a number of clinical studies, the physiological ranges differ greatly. While compared to some studies, the median concentrations for both biomarkers seem to be higher in our cohort than in age-matched reference subjects [72, 73], both biomarkers were within normal range according to other references [74, 75]. Whether these differences compared to healthy women are due to the g*BRCA1/2* mutation or other factors is currently unclear and subject to further research.

With our analysis, we focused on the potential influences of exercise and nutritional habits on biomarker serum levels. While highlighting the preventive potential of health habits in reaching favorable biomarker constellations, this approach does not provide prognostic value. So far, there are ambiguous findings whether OPG and sRANKL actually are prognostic markers for BC risk. While Vik et al. reported a significantly inverse association between OPG and *BRCA1/2*-associated BC risk [76], Kotsopoulos and colleagues did not find evidence for an association between plasma OPG [77] or sRANKL [78] levels and BC risk. However, the validity of their study might be limited as plasma samples were collected in the late 1990s and hence stored over 20 years, possibly altering protein concentrations. Furthermore, Kotsopoulos et al. postulate that a *single* measurement of OPG and sRANKL is able to predict the BC risk in the future. This stands in contrast to our findings, suggesting the biomarkers are dynamic and responsive to lifestyle changes also in the short term.

## Conclusion

The aim of our analysis was to objectively demonstrate the effects of a controlled lifestyle intervention, including physical activity and adherence to a MedD, on serum concentrations of OPG and sRANKL.

Showing that changes in health habits can lead to favorable changes in biomarker levels, especially OPG may provide a first step in research for establishing clinical risk factors as well as potential preventative options for g*BRCA1/2* mutation carriers with regards to the incidence of hereditary BC disease.

## Supplementary Information

Below is the link to the electronic supplementary material.

Supplementary file1 (DOCX 19 kb)

## Data Availability

The datasets generated during and/or analyzed during the current study are available from the corresponding author on reasonable request. Individual participant data will be available after de-identification (text, tables, figures). The study protocol has been published elsewhere.

## Abbreviations

ARA: Arachidonic acid
BC: Breast cancer
BMI: Body mass index
CG: Control group
CLA: Linoleic acid
CPET: Cardiopulmonary exercise testing
DHA: Docosahexaenoic acid
DPA: Docosapentaenoic acid
ELISA: Enzyme-linked immunosorbent assay
EPA: Eicosapentaenoic acid
ETA: Eicosatrienoic acid
FDR: False discovery rate
g*BRCA1/2*: Germline mutation in the breast cancer genes *BRCA1* or *BRCA2*
HRT: Hormone replacement therapy
IG: Intervention group
IQR: Interquartile range
LIBRE-1: Pilot study of the lifestyle intervention study in women with hereditary breast and ovarian cancer
LIBRE-2: Lifestyle intervention study in women with hereditary breast and ovarian cancer
MEDAS: Mediterranean diet adherence screener
MedD: Mediterranean diet
NF: Nuclear factor
OC: Ovarian cancer
OPG: Osteoprotegerin
PUFA: Polyunsaturated fatty acids
RANK: Receptor activator of nuclear factor κB
RBCM: Red blood cell membranes
REF: Reference number
SE: Study entry
sRANKL: Soluble receptor activator of nuclear factor κB ligand
V1: Study visit 3 months after study entry in LIBRE-1
VO_2_ peak: Maximal oxygen uptake in ml/min/kg

## Code availability

Statistical analysis was conducted using GraphPad Prism version 8.4.3. for MacOS (GraphPad Software, San Diego, California, USA).
