# Supplementary material for: Physical activity and Mediterranean diet as potential modulators of osteoprotegerin and soluble RANKL in gBRCA1/2 mutation carriers: results of the lifestyle intervention pilot study LIBRE-1
Source: Breast Cancer Res Treat. 2021 Sep 27;190(3):463–75. doi: 10.1007/s10549-021-06400-7 (PMC8558155; doi:10.1007/s10549-021-06400-7)
Supplement: Supplementary file 1 — Supplementary file1 (DOCX 19 kb) [file 10549_2021_6400_MOESM1_ESM.docx]

**Supplementary Table 1** Association between changes in sRANKL and OPG and changes in n-3, n-6 and n-9 fatty acids during the course of the three-month intervention phase (ΔV1-SE).

|  | sRANKL (IG: n=17, CG: n=19) | | | OPG (IG: n=14, CG: n=12) | | |
| --- | --- | --- | --- | --- | --- | --- |
|  | Spearman r | 95%CI | p (q) value | Spearman r | 95%CI | p (q) value |
| RBCM PUFA | | | | | | |
| n-3^a^ | -0.1067 | -0.4332-0.2445 | 0.5417 (0.8372) | 0.5788 | 0.2354-0.7937 | 0.0019 (**0.0476**) |
| n-6^b^ | -0.0913 | -0.4205-0.2591 | 0.6019 (0.8527) | -0.0735 | -0.4577-0.3338 | 0.7212 (0.8750) |
| n-9^c^ | -0.1591 | -0.4755-0.1938 | 0.3613 (0.6465) | -0.2007 | -0.5541-0.2140 | 0.3256 (0.6343) |
| EPA 20:5  (n-3) | -0.0726 | -0.4048-0.2766 | 0.6788  (0.8750) | 0.5624 | 0.2123-0.7845 | 0.0028 (**0.0476**) |
| DPA 22:5  (n-3) | 0.0087 | -0.3346-0.35 | 0.9605 (0.9774) | 0.4906 | 0.1156-0.7432 | 0.0109 (0.0741) |
| DHA 22:6  (n-3) | -0.1283 | -0.4508-0.2239 | 0.4627  (0.7491) | 0.4270 | 0.3545-0.7049 | 0.0296 (0.1258) |
| ARA 20:4  (n-6) | 0.0092 | -0.3341-0.3505 | 0.9580  (0.9774) | -0.2732 | -0.6050-0.1396 | 0.177 (0.4629) |
| Oleic acid 18:1 (n-9) | -0.0832 | -0.4137-0.2667 | 0.6347 (0.8632) | -0.2000 | -0.5536-0.2146 | 0.3273 (0.6343) |
| Plasma PUFA | | | | | | |
| n-3^d^ | -0.0293 | -0.3112-0.3632 | 0.8651 (0.9774) | 0.3074 | -0.1028-0.6282 | 0.1267 (0.3916) |
| n-6^e^ | 0.3449 | 0.0084-0.6113 | **0.0396** (0.1496) | -0.5296 | -0.7659- -0.1672 | **0.0054** (0.0612) |
| n-9^f^ | -0.21 | -0.5113-0.1372 | 0.2189 (0.4962) | 0.07282 | -0.3344-0.4572 | 0.7237 (0.8750) |
| EPA20:5  (n-3) | 0.0049 | -0.3332-0.3418 | 0.9774  (0.9774) | 0.06667 | -0.3399-0.4523 | 0.7463 (0.8750) |
| DPA22:5 (n-3) | 0.2108 | -0.1364-0.5119 | 0.2171  (0.4962) | 0.1631 | -0.2508-0.5265 | 0.4260 (0.7242) |
| DHA22:6  (n-3) | -0.02 | -0.3552-0.3196 | 0.9075  (0.9774) | 0.3737 | -0.02807-0.6715 | 0.06 (0.2040) |
| ARA20:4  (n-6) | 0.0963 | -0.2493-0,4291 | 0.5765  (0.8522) | -0.1966 | -0.5511-0.2180 | 0.3358 (0.6343) |
| Oleic acid 18:1 (n-9) | -0.3683 | -0.6278-0.0352 | **0.0271** (0.1258) | 0.5070 | 0.1371-0.7528 | **0.0082** (0.0695) |

Correlation analyses using Spearman rank (r) between changes (ΔV1-SE) in OPG and sRANKL and changes in n-3, n-6 and n-9 fatty acids (FA) measured in red blood cell membranes (RBCM) and plasma during a three-month intervention phase. A post-hoc test was used to adjust for multiple testing according to Benjamini and Hochberg with Q at 5% (threshold: p<0.0031) based on p-values in supplementary table 1 and IG/CG subgroup analysis, resulting in q-values, as shown in brackets. OPG, osteoprotegerin; PUFA, polyunsaturated fatty acids; sRANKL, soluble receptor of nuclear factor κB ligand; V1, three months after SE.

^a^ Sum of eicosatrienoic acid (ETA, 20:3 n-3), eicosapentaenoic acid (EPA, 20:5 n-3), docosapentaenoic acid (DPA, 22:5 n-3) and docosahexaenoic acid (DHA, 22:6 n-3).

^b^ Sum of linoleic acid (CLA, 18:2 n-6) and arachidonic acid (ARA, 20:4 n-6).

^c^ Sum of oleic acid (18:1 n-9), gondoic acid (20:1 n-9) and nervonic acid (24:1 n-9)

^d^ Sum of alpha-linolenic acid (18:3 n-3), EPA, DPA and DHA.

^e^ Sum of CLA, gamma linoleic acid (18:3 n-6), eicosadienoic acid (20:2 n-6), dihomo-gamma-linolenic acid (20:3 n-6), ARA

^f^ Sum of erucic acid (22:1 n-9), eicosadienoic acid (20:2 n-9), mead acid (20:3 n-9)
